# Exercise and Protein Intake: A Synergistic Approach against Sarcopenia

**DOI:** 10.1155/2017/2672435

**Published:** 2017-03-21

**Authors:** Anna Maria Martone, Emanuele Marzetti, Riccardo Calvani, Anna Picca, Matteo Tosato, Luca Santoro, Angela Di Giorgio, Antonio Nesci, Alex Sisto, Angelo Santoliquido, Francesco Landi

**Affiliations:** ^1^Department of Geriatrics, Neurosciences and Orthopedics, Catholic University of the Sacred Heart, Rome, Italy; ^2^Department of Medicine, Catholic University of the Sacred Heart, Rome, Italy

## Abstract

Sarcopenia, the age-dependent loss of muscle mass and function/strength, is increasingly recognized as a major risk factor for adverse outcomes in frail older people. As such, the skeletal muscle is a relevant target for interventions aimed at preventing or postponing the occurrence of negative health-related events in late life. The association among physical inactivity, insufficient intake of energy and protein, and poor muscle health in older adults suggests that physical exercise and targeted nutritional supplementation may offer substantial therapeutic gain against sarcopenia and its negative correlates. This view is supported by observational studies as well as by small-scale clinical trials. In this review, we summarize the available evidence on the beneficial effects of behavioral interventions on sarcopenia. We also briefly describe how the knowledge gathered so far has been used to design the “Sarcopenia and Physical fRailty IN older people: multicomponenT Treatment strategies” (SPRINTT) project. The randomized clinical trial conducted within SPRINTT will provide robust evidence on the effectiveness of exercise and nutrition at preventing negative outcomes associated with sarcopenia and physical frailty.

## 1. Introduction

The aging process is accompanied by multisystem derangements that ultimately deplete the homeostatic capacity of the organism as a whole. The progressive loss of muscle mass and strength/function is one of the most noticeable correlates of aging, evident across multiple species [1]. This condition, referred to as sarcopenia [2], has a multifactorial origin, involving lifestyle habits, disease triggers, and age-dependent biological changes (e.g., chronic inflammation, mitochondrial abnormalities, loss of neuromuscular junctions, reduced satellite cell number/function, and hormonal alterations) [3].

Muscle decay is advocated as a true biomarker of aging, able to distinguish, at the clinical level, biological from chronological age [1]. Not surprisingly, the “muscle aging” phenomenon, as a paradigm for exploring the aging process as a whole, has attracted remarkable attention in the field of biogerontology [1]. At the same time, the recognition of sarcopenia as a major determinant of adverse health outcomes has ignited growing interest among researchers in the attempt of devising interventions to halt or reverse muscle aging [4].

The lack of a univocal operational definition of sarcopenia represents a major limitation in the field. Originally described solely as the age-related loss of muscle mass [5], it is now widely acknowledged that sarcopenia encompasses both quantitative (i.e., mass) and qualitative (i.e., strength and/or function) declines of skeletal muscle [6]. Yet, depending on the tools used for the assessment of muscle mass and function and the reference values considered, the resulting phenotypes are only partly overlapping [7]. As a consequence, sarcopenia has not yet been incorporated in everyday clinical practice nor is its underlying pathophysiology fully understood, which impacts the discovery of meaningful biological targets for interventions.

In such a scenario, physical exercise and adequate protein and energy intake are to date the only strategies of proven efficacy (and safety) to improve muscle health across ages [8]. It is speculated that the combination of the two interventions might convey much larger benefits than those achievable through the administration of either of them. Indeed, the synergy between exercise and diet may tackle sarcopenia in multiple aspects, possibly maximizing the effects brought about by each of the two components [9].

## 2. Protein and Muscle: An Intimate Liaison

Muscle mass is controlled by complex interactions of multiple factors; nonetheless, the dynamic balance between protein synthesis and breakdown is a major determinant of it [10]. Indeed, meaningful losses or gains in muscle mass and quality result from sustained variations in muscle protein synthesis, breakdown, or the combination of both processes. Whether sarcopenia primarily results from diminished basal protein synthetic rates or blunted muscle anabolism in response to anabolic stimuli is yet to be established.

**Figure 1:**
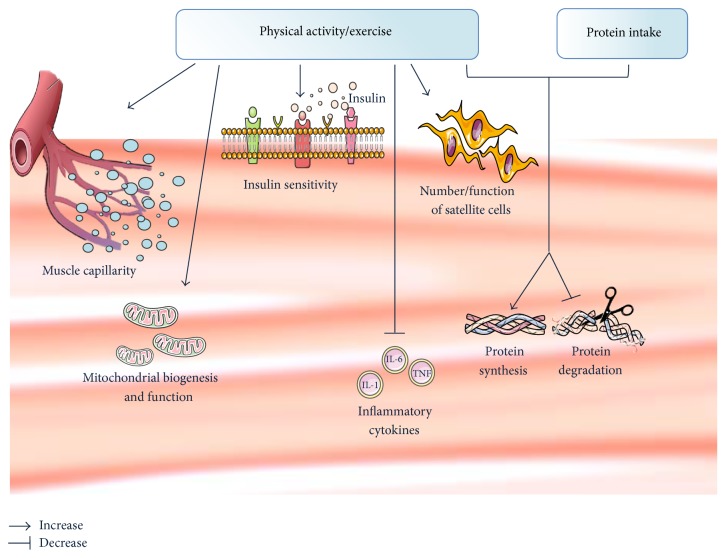
Scheme illustrating major effects of physical activity/exercise and protein intake on muscle physiology. IL: interleukin; TNF: tumor necrosis factor.

Muscle protein synthesis is regulated by several anabolic stimuli, including physical activity and food ingestion. Essential amino acids (EAAs) are the most important nutritional inputs for protein synthesis. In this respect, leucine is considered the primary nutritional regulator of muscle protein anabolism [11], due to its ability to trigger the mammalian target of rapamycin (mTOR) pathway and inhibit the proteasome (Figure 1) [11]. It is worth noting that aged muscles show a reduced anabolic response to low doses (e.g., less than 10 g) of EAAs [12]; yet, higher doses (e.g., 10–15 g, with at least 3 g of leucine) are sufficient to induce a protein anabolic response comparable to that observed in younger adults [12]. Therefore, it is recommended that older persons consume protein sources with higher proportions of EAAs (i.e., high-quality proteins), such as lean meat and other leucine-rich foods (e.g., soybeans, peanuts, cowpea, and lentils) [13].

Recently, consensus and opinion articles as well as small-scale clinical trials have emphasized that protein intake above the current recommended dietary allowance (RDA; 0.8 g/kg/day) may be requested to maintain muscle health in late life [14–17]. It appears therefore appropriate to promote protein intake of 1.0–1.2 g/kg/day, while 1.2–1.5 g/kg/day of protein may be required in older adults with acute or chronic diseases [10, 16, 17]. Finally, older people with severe illnesses or overt malnutrition may need as much as 2.0 g/kg/day of protein [17].

Closely related to the amino acid composition of dietary proteins is their absorption kinetics. Indeed, the speed of protein digestion and amino acid absorption by the gut impacts postprandial protein deposition as well as protein breakdown [18]. Noticeably, in young individuals, slowly digested proteins (e.g., casein) produce greater protein retention than those that are digested faster (e.g., whey) [18]. A divergent pattern has been observed in older persons, such that the postprandial protein synthesis appears to be stimulated to a greater extent by whey proteins as opposed to casein [19, 20]. Although the concept of “fast” versus “slow” protein has been questioned [21], the evidence accumulated thus far is sufficiently robust to recommend the prescription of fast-digested protein supplements to improve muscle health in old age [17].

With regard to the source of dietary protein, no conclusive evidence is available about the possible differential effects of animal-derived versus plant-based proteins. It should however be considered that animal protein sources generally contain greater amounts of EAAs and are more digestible than those of vegetal origin [22]. Furthermore, plant-based proteins undergo greater splanchnic extraction and subsequent urea synthesis than animal-derived proteins [22]. Recently, the consumption of large amounts of soy proteins, as a strategy to overcome their lower anabolic potency, has been shown to stimulate muscle protein synthesis in older men to a smaller degree than isolated whey proteins, both at rest and following exercise [23]. Such a difference has been attributed to the fact that soy proteins are directed more toward oxidation rather than being used for de novo muscle protein synthesis. In addition to proteins, meat contains a vast array of biologically active compounds (e.g., creatine, carnitine, iron, and cobalamin), all of which may favorably influence muscle physiology [24]. Based on the current evidence, the consumption of lean meat 4-5 times a week should therefore be recommended to maintain muscle health in advanced age.

Finally, the formulation of protein sources may have an important effect on protein digestion, amino acid absorption, and muscle anabolism. For instance, Conley et al. [25] showed that older adults achieved higher plasma amino acid concentrations after ingestion of a liquid meal replacement product as compared with an energy- and macronutrient-matched solid replacement. Whether specific physical properties of protein sources may be harnessed to obtain therapeutic gain in the context of sarcopenia warrants further investigation.

## 3. Exercise: Much More Than Mechanical Stimulation

Physical activity refers to any level of bodily movement that results from skeletal muscle activation and leads to an increase in energy expenditure [26]. Exercise, instead, is a planned, structured, repetitive activity aimed at improving fitness [26]. Physical activity and exercise represent, to date, the most effective interventions for the promotion of healthy aging [27]. With regard to the skeletal muscle, exercise mitigates several deleterious effects of aging, including impaired insulin sensitivity, mitochondrial dysfunction, acceleration of myonuclear apoptosis, and inflammation (Figure 1) [28].

Among the various types of training, low-intensity endurance exercise increases aerobic fitness by improving skeletal muscle oxidative capacity and cardiovascular function. The increased muscle capillarity allows matching the greater requests for mitochondrial oxygen flux [29, 30]. Enlargement of the muscle mitochondrial compartment is typically observed during endurance exercise, especially in untrained persons (Figure 1). Conversely, the muscle fiber cross-sectional area is relatively unaffected by this type of training [31]. High-load resistance exercise, instead, impacts both fiber cross-sectional area and muscle function (strength and power), mainly through increasing the number and size of fast-twitch fibers (i.e., types IIA and IIX) [30]. In response to different exercise protocols, muscle cells modulate the expression of specific proteins pertaining to mitochondrial biogenesis and function, such as peroxisome proliferator activated receptor gamma coactivator-1 alpha (PGC-1*α*) and muscle fatty acid binding protein (mFABP) [28]. In particular, PGC-1*α*, by activating several transcription factors, orchestrates mitochondrial biogenesis, while mFABP is involved in fatty acid utilization for mitochondrial energy production [28].

The activation and recruitment of satellite cells are believed to represent a major adaptation to chronic exercise (Figure 1). The incorporation of new nuclei from satellite cells into existing muscle fibers increases the number of “myonuclear domains” (i.e., the anatomical and functional units made up by a myonucleus and the surrounding volume of sarcoplasm) and, therefore, the fiber cross-sectional area [32]. Satellite cell activation is influenced by a number of factors, including age, nutritional status, and type and intensity of physical exercise. During physical exercise, myofibers release hormones and inflammatory mediators able to activate satellite cells [33], ultimately increasing the number of nuclei incorporated in muscle fibers.

Downregulation of systemic inflammation is an additional mechanism whereby exercise positively impacts muscle physiology. Studies have shown a decrease of circulating levels of several inflammatory mediators in older adults engaged in exercise interventions (both aerobic and resistance training) [34]. Such an adaptation has been correlated with improvements in muscle mass and physical performance [35–37]. Furthermore, downregulation of tumor necrosis factor alpha (TNF-*α*) mediated myonuclear apoptosis has been observed in senescent rodents performing treadmill exercise, in conjunction with gains in muscle mass and strength [38].

Collectively, available evidence indicates that regular physical exercise positively affects muscle physiology through local and systemic effects. As discussed in the next section, it is plausible that the combination of exercise and targeted nutrition interventions may be a more valuable strategy to manage sarcopenia.

## 4. Protein Supplementation and Exercise: A Plausible, Magnificent Strategy

The intensity, duration, and mode of the activity performed as well as the nutritional status and diet (in particular daily protein intake) markedly influence skeletal muscle mass, strength, and metabolism [28, 39]. However, in older people, muscular responses to a single anabolic stimulus may be blunted compared with young persons, possibly reflecting differences in the physiological reserve across life stages [28]. The combination of exercise with increased protein intake seems to be the most plausible strategy to overcome such an issue (Figure 1). This view has been embraced by several scientific societies as well as experts in the fields of muscle aging, nutrition, and physiology [17, 40, 41]. In this regard, Tieland et al. [42] showed that 24-week protein supplementation combined with resistance training increased muscle mass, strength, and physical performance in frail elderly participants. The combination of protein plus exercise allowed for larger gains in muscle mass relative to exercise alone [43].

As highlighted by Cruz-Jentoft et al. [40], assessing the possible synergistic effect of a multicomponent intervention (exercise plus nutrition/protein supplementation) on muscle properties is a challenging task. Ideally, studies should use at least four arms (i.e., exercise, nutrition, both, and neither of them), should be conducted for a sufficient time, and should enroll an adequately large population to appreciate the differential responses elicited on “anabolic resistant” aged muscle. Another critical aspect to consider is the timing of “administration” of exercise and nutrition. Muscle performance and feeding patterns are influenced by and affect circadian rhythms [43, 44] that could themselves be altered over the course of aging [45]. In addition, muscle protein synthesis fluctuates quite largely during the day and is influenced by several factors, including total energy/carbohydrate intake, timing of protein intake, and functional and training status [46]. In this respect, it should be considered that the existence of an optimal time window for protein consumption in relation to exercise is still a matter of debate. Traditionally, it is assumed that muscle is responsive to nutrient ingestion, especially amino acids, for up to three hours following resistance exercise [47]. However, inconsistent results in timing and total protein effects on muscle metabolism and lean body mass accretion have been reported, especially in older persons engaged in chronic resistance exercise programs [48]. Moreover, as elegantly highlighted by Simmons et al. [46], the effect of the timing of protein intake in conjunction with exercise may be biased by the methodologies used to measure muscle protein synthesis. That said, the identification of a theoretical anabolic time window for nutrient/protein ingestion would be highly relevant to older adults. Indeed, the consumption of protein at the time when the muscle anabolic responsiveness is maximal would allow for reducing the overall protein load and maintaining fat-free mass during periods of either increased energy expenditure or limited protein intake (e.g., exercise, chronic and acute disease conditions) [48].

## 5. The SPRINTT Clinical Trial: Testing Exercise and Nutrition in the “Real World”

Despite the encouraging findings obtained by observational studies and small clinical trials, the effectiveness of combining physical exercise and dietary protein intervention at preventing adverse health outcomes in older adults has not yet been conclusively established. The ongoing “Sarcopenia and Physical fRailty IN older people: multicomponenT Treatment strategies” (SPRINTT) project has been specifically designed to provide clinicians and health authorities with all the evidence necessary to promote practical strategies for older adults at risk of physical disability [49]. With an expected sample size of 1,500 participants followed up for an average of 24 months, SPRINTT is the largest and longest clinical trial designed to test the effectiveness of a multimodal strategy at preventing mobility disability in older adults with physical frailty and sarcopenia (PF&S) [49, 50]. The PF&S condition is defined by the cooccurrence of low muscle mass (according to the cut points recommended by the Foundation for the National Institute of Health Sarcopenia Project [51]) and reduced physical performance, operationalized as a summary score on the Short Physical Performance Battery [52] between three and nine. Older adults with PF&S are randomized to either a multicomponent intervention (MCI), involving structured physical activity, nutritional counseling/dietary intervention, and an information and communication technology (ICT) intervention, or a Healthy Aging Lifestyle Education (HALE) program [49]. The physical activity program has been taken and modified from the Lifestyle Interventions and Independence for Elders (LIFE) study [53], given its full safety profile and efficacy in preventing mobility disability in at-risk older persons. As part of the ICT component of the MCI, actimetry data are collected through a dedicated device in order to monitor adherence to physical activity and provide personalized feedback/tips to the participant.

The nutritional component of the SPRINTT study has been designed to maximize the benefits of physical activity. As previously discussed, nutrition represents an important and potentially modifiable factor that impacts muscle health and the frailty status of older people [8, 54]. As such, not only is nutrition involved in the direct assessment of frailty, but also it may play a role in the definition of interventions aimed at restoring robustness and contrasting sarcopenia [8, 55]. In SPRINTT, the multifactorial properties of nutrition in bolstering the beneficial effect of physical activity on PF&S are exploited through the combination of nutritional assessments and personalized dietary recommendations. SPRINTT broadly aims at achieving two predefined nutritional targets: (1) total daily energy intake of 25–30-kcal/kg body weight [56] and (b) average daily protein intake of at least 1.0–1.2 g/kg body weight [17].

## 6. Conclusions

The preservation of muscle mass and function is increasingly recognized as a crucial factor for promoting healthy aging and improving quality of life. As such, sarcopenia represents an ideal target for interventions aimed at preventing or postponing the occurrence of negative health-related events in late life. At present, multicomponent lifestyle interventions, involving the combination of exercise and nutrition (in particular, adequate protein intake), are the only “fountain of youth” available to achieve healthy aging. High-quality clinical trials are needed to identify the type, modes, and duration of multidomain interventions that maximize the health benefits in advanced age.
